# Teamwork of clinical teachers in postgraduate medical training

**DOI:** 10.1007/s40037-016-0286-0

**Published:** 2016-07-18

**Authors:** Irene Arida Slootweg

**Affiliations:** Professional Performance Research group, Center for Evidence-Based Education (CEBE), Academic Medical Center, Amsterdam, The Netherlands

**Keywords:** Teamwork, Leadership, Postgraduate medical training, Faculty development

## Abstract

Teamwork among clinical teachers is essential for continuous improvement of postgraduate medical training. This thesis deconstructs teamwork in four studies, mostly based on qualitative research approaches and one study utilizes mixed methods. We found that clinical teachers do train residents, but individually rather than as a team. The programme directors as leaders focus more on teaching activities than on the collective ambition and mutual engagement of clinical teachers. During the teaching meetings, mistakes and conflicts are mainly discussed in a general sense and are often neither directed at the individual, nor result-oriented. A valid evaluation instrument is constructed to improve teamwork.

## Introduction

This series of four studies explores teamwork among clinical teachers in postgraduate medical training. The importance of clinical teachers sharing information about teaching, supervision and feedback has been stressed in the literature [1]. If clinical teachers operate as a team they communicate about collective professional ambition, promote a constructive learning climate and anticipate on the continuously changing context within postgraduate medical training. Jointly carrying out an objective and transparent workplace-based assessment represents a significant challenge for clinical teachers [1–3]. In the literature on the development of clinical teachers’ teaching and assessment qualities, pitfalls have been described, such as the lack of agreement among teachers about what constitutes satisfactory resident performance [4]. We know that in general clinical teachers favour a collegial and loyal attitude in working together, also with colleagues who hold a fundamentally different opinion about the residents [5]. It remains unknown, however, what teamwork specifically among clinical teachers comprises, for instance, how teamwork is being evaluated, how clinical teachers communicate with each other and how leadership by programme directors influences teamwork. To deconstruct teamwork of clinical teachers, we created two overarching research questions: *What is the nature of teamwork among clinical teachers? *and* What is the role of leadership in supporting the teamwork of clinical teachers?* The aim is to contribute to the development of knowledge on teamwork and leadership and to offer guidelines for clinical teachers.

## Methodology

The thesis as a whole takes a constructivist approach to knowledge and to the reality around us. In these studies most knowledge was constructed together with the participants [6]. For the development of an evaluation instrument a post-positivistic paradigm was applied, whereby the truth of teamwork was sought at a specific moment in time. The first study explored how clinical teachers themselves talk about their experience of teamwork in postgraduate medical training. The lived experiences of clinical teachers were studied using six focus groups (*n* = 50) within a qualitative, phenomenological approach. The second study continued to build on the findings of the first study, in order to develop a valid and reliable instrument to evaluate the teamwork of clinical teachers. We conducted a mixed methods study with a Delphi procedure (*n* = 40 experts), pilot tested the initial instrument (*n* = 116 teaching teams), and performed various statistical analyses, such as principal component analysis, estimation of internal consistency reliability coefficient and computation on the number of evaluations needed to obtain reliable estimates. The mixed methods approach enabled us to identify measurable items based on the literature and the opinion of the experts, and to develop a reliable instrument based on investigating the psychometric qualities of the tool. We intended to develop an instrument to facilitate clinical teachers in making the shift from discussing the *teamwork constructs *to contemplating the *results of evaluation* as a first step in improving teamwork. The third study clarified the role of programme directors in the teaching team using a phenomenographic, qualitative approach in which ‘strategic leadership’ was used as the theoretical framework. This framework defines three key features of strategic leadership: (i) designing the organization, (ii) developing collectivity by providing information and (iii) collaborative learning. Using interviews (*n* = 14) programme directors’ experiences with strategic leadership were examined [7]. The fourth and final study added depth to the insights on teamwork by studying team communication during a formal teaching meeting. For data collection and analyses we used the behaviours of speaking up as theoretical framework: asking questions, seeking feedback and help, discussing mistakes and sharing information [2]. It was a modified ethnographic study applying observations of the formal meetings, followed by interviews with the programme directors (*n* = 10 teaching teams), based on audio fragments of the meeting.

## Results

Clinical teachers’ perceptions on teamwork, explored in the first study, resulted in the description of seven themes of teamwork [8]. Building on these findings we developed an instrument (called TeamQ) to evaluate teamwork in clinical teaching teams for residency training. Data collection among 132 clinical teams and statistical analysis resulted in eight factors of teamwork of clinician teachers: *task expertise, team expertise, decision-making, team leadership, feedback culture, team results, residents’ engagement *and *residents’ empowerment*. A valid, reliable and feasible web-based evaluation instrument with ideal-formulated items and a 5-point Likert-scale is now available for clinical teachers to picture their strengths and weaknesses of teamwork in postgraduate medical training [9].

In the study on leadership, we found programme directors prefer to lead their teaching teams in two of the key features of strategic leadership: designing the organization and providing information to develop collectivity [10]. Programme directors invest little in collective goals and strategy and they have less experience with leading for collaborative learning. Additionally, we identified four leadership profiles within which the experience of the programme directors can be sorted out: *‘captains’,‘carers’, ‘professionals’ and ‘team players’*. In the study on team communication we found that clinical teachers exhibit most of the behaviours of speaking up. They demonstrate strongly providing information instead of sharing information. Mistakes and conflicts are mainly discussed in a general sense and are often neither directed at the individual nor adequately result-oriented. We identified three factors influencing the communication between clinical teachers during the formal educational meetings (i) *relational (power, leadership, handling conflicts, feeling safe), *(ii) *cultural (history, feedback – and meeting culture), *(iii) *professional (discipline-specific, commitment of teaching, contribution of residents and personal traits)* [11].

## Discussion

The answer to the overall questions: *What is the nature of teamwork among clinical teachers? *and* What is the role of leadership in supporting the teamwork of clinical teachers?* breaks down into four main findings. First, clinical teachers pay little or no attention to practising and developing teamwork qualities. Individual teamwork qualities refer to the knowledge, skills and attitudes that clinical teachers need in order to be able to work together effectively [12]. Second, the team process is primarily an ‘action process’ in which clinical teachers pay little or no attention to the ‘transition processes’ and the ‘interpersonal processes’ [13]. The ‘action process’ was primarily focused on providing information and implementing teaching tasks. The studies show little or no evidence of the ‘transition processes’ which focus on mission analysis and on formulating strategic goals. The ‘interpersonal processes’ do occur, these seem to be aimed specifically at avoiding conflicts and preserving harmonious relations. Third, the clinical teachers certainly show individual results in the training of residents, although these results are hardly presented as a joint team. Clinical teachers carry out independent teaching activities individually, but these activities are not well-embedded in a collective ambition or in a system of mutually influencing activities. This may weaken the team result [14]. Fourth, the programme directors’ support for teamwork to safeguard the quality of the postgraduate medical training appears to be limited. The programme directors invest very little in the collective, professional ambition of clinical teachers. There is little or no visible evidence of a programme director being given or taking the opportunity to learn, how to be a teaching team [2].

A particular strength of this series of studies is the overall design. All studies were designed consecutively and each study informed the next, which strengthens the findings and consistency. Further strengths are the number of different innovative research designs that were applied in these studies. A limitation is that this series of studies was based on perceptions and behaviours of clinical teachers and programme directors only. Perceptions and behaviours of the residents are not taken into account, although they are – to a greater or lesser extent – members of a teaching team too. It would be useful to study the experience of residents with teamwork in clinical teaching teams. A logical step in follow-up research would be to pay attention to the dynamics between clinical teachers and the programme director in developing team learning in a clinical teaching team [15]. The practical implication of this body of knowledge is its contribution to raising awareness of the importance of teamwork in postgraduate medical training among clinical teachers. This is the first step in creating the urgency to apply and develop teamwork qualities (the belief in teamwork) and to allow clinical teachers to experience the positive effects of teamwork. The programme directors can reflect on their personal leadership qualities on the basis of our four profiles and can develop their leadership skills, at the same time as clinical teachers develop team learning. In order to practice accountability and to monitor and improve, their teamwork, clinical teaching teams should regularly evaluate their teamwork using the TeamQ as part of the quality assurance of postgraduate medical training.

## Conclusion

The nature of teamwork of clinician teachers can be characterized as follows: clinical teachers do train residents passionately, but not as a team with collective ambitions, shared values, common goals and clear team results. If learning and working as a team were to receive more attention, the clinical teachers and the programme directors as leaders may give a positive impulse to the quality of postgraduate medical training.

### Tips.

Choose a topic that makes your heart beat faster. Consider the PhD journey as a personal learning development to discover your own research identity.
